# Molar loss and powder diet leads to memory deficit and modifies the mRNA expression of brain-derived neurotrophic factor in the hippocampus of adult mice

**DOI:** 10.1186/s12868-016-0319-y

**Published:** 2016-12-05

**Authors:** Yosuke Takeda, Hiroshi Oue, Shinsuke Okada, Akira Kawano, Katsunori Koretake, Makoto Michikawa, Yasumasa Akagawa, Kazuhiro Tsuga

**Affiliations:** 1 Department of Advanced Prosthodontics, Hiroshima University Graduate School of Biomedical and Health Sciences, Hiroshima, Japan; 2 Department of Biochemistry, Nagoya City University Graduate School of Medical Sciences, Nagoya, Japan

**Keywords:** Molar loss, Powder diet, Memory deficit, Brain-derived neurotrophic factor

## Abstract

**Background:**

It is known that tooth loss is known to be a risk factor for Alzheimer’s disease and soft diet feeding induces memory impairment. Recent studies have shown that brain-derived neurotrophic factor (BDNF) is associated with tooth loss or soft diet in young animal model, and that BDNF expression is decreased in patients with Alzheimer’s disease. However, single or combined effect of tooth loss and/or soft diet on brain function has not fully understood. Here we examined the effect of molar loss and powder diet on memory ability and the expression of BDNF mRNA in the hippocampus of adult C57BL/6J mice. Twenty eight-weeks-old C57BL/6J mice were divided into intact molar group and extracted molar group. They were randomly divided into the I/S group (Intact upper molar teeth/Solid diet feeding), the E/S group (Extracted upper molar teeth/Solid diet feeding), the I/P group (Intact upper molar teeth/Powder diet feeding), and the E/P group (Extracted upper molar teeth/Powder diet feeding). The observation periods were 4 and 16-week. To analyze the memory ability, the step-through passive avoidance test was conducted. BDNF-related mRNA in the hippocampus was analyzed by real-time polymerase chain reaction (RT-PCR).

**Results:**

At 4 weeks later, we performed memory test and isolated brains to analyze. There were no differences in memory function and BDNF mRNA level between these four groups. However, at 16 weeks later, E/S and E/P group showed memory impairment, and decreased level of BDNF mRNA. Whereas, the powder diet had no effect on memory function and BDNF mRNA level even at 16 weeks later.

**Conclusions:**

These results suggest that the effect of molar loss and powder diet on memory function and BDNF mRNA levels were different, molar loss may have a greater long-term effect on memory ability than powder diet does.

## Background

Maintenance of healthy dental conditions and mastication has been believed to play a crucial role not only in oral function, but also in systemic conditions and brain function [1–3]. Retrospective studies have reported the relationship between oral health and cognitive impairment [4–6]. Community-dwelling persons with tooth loss are more likely to have impaired cognitive test performance, with those older than 45 years being more significantly affected [5]. Another study reported that the possession of fewer teeth in adulthood was significantly associated with incident of dementia [6] or tooth loss has been suggested as a possible epidemiological risk factor for Alzheimer’s disease (AD) [7]. Additionally, animal studies suggested that impairment of oral condition, including tooth loss, could lead to memory impairment [8–10].

Brain-derived neurotrophic factor (BDNF), a member of the neurotrophic family, is widely expressed in the central nervous system and plays an important role in the regulation of hippocampal learning and memory processes [11]. Also, BDNF is a key molecule involved in the structural and functional plasticity of the hippocampal synapse. In addition, decreased BDNF levels may result in decreased hippocampal neurogenesis; BDNF expression is decreased in patients with AD and Parkinson’s disease [12–14]. BDNF actions include activation of insulin receptor substrate-1 (IRS-1/2), Pl-3K, and protein kinase B (Akt). BDNF binds to tyrosine kinase B (TrkB), which is a high affinity receptor of BDNF and activates a signal transduction cascade [15]. BDNF and its tyrosine kinase receptor, TrkB, are expressed in hypothalamic nuclei associated with satiety and locomotor activity [26].

Some reports suggest that BDNF might be associated with molar loss or soft diet [16, 17]. TrkB was also reduced in association with molar loss [16, 17]. Molar loss is associated with a reduction in the number of pyramidal cells in the hippocampus of AD model mice [9]. Liquid diet, with reduced mastication, was associated with reduced memory ability and BDNF levels in a mouse model [17]. In clinical practice, particularly with elderly patients, molar loss can lead to the reduction of masticatory function, and those who have few teeth tend to prefer soft diets [18]. Previous studies, however, examined the effect of either molar loss or soft diet on BDNF level at young age. Therefore, after maturation, the effect of molar loss and soft diet on cognitive function has not been fully understood. Analysis of these effects in adulthood is important, because hippocampal neurogenesis has been reported to occur not only in childhood but also in adulthood in both humans and animals [19, 20].

Therefore, we focused on the combination of molar loss and powder diet could affect memory ability or BDNF-related mRNA in the hippocampus. Our results could clarify the effect of molar loss and diet texture on cognitive function of adult mice.

## Methods

### Ethics

The protocol of this animal study was approved by the Research Facilities Committee for Laboratory Animal Science at the Hiroshima University School of Medicine and performed in accordance with the current version of the Japan Law on the Protection of Animals.

### Animals

**Fig. 1:**
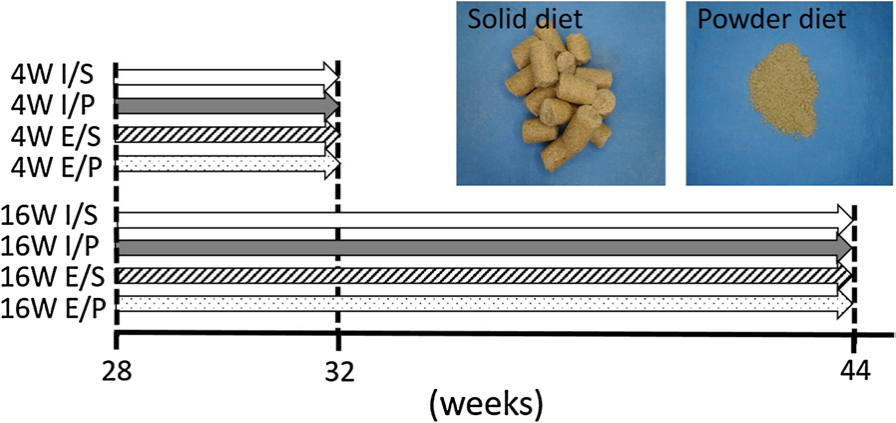
Time schedule of this experiment. Mice were randomly divided 4 groups for each experimental period (n = 12). At 32 and 44-weeks, behavioral test was performed in each 4 and 16 W group. *I* Intact upper molar tooth. *E* Extracted upper molar tooth. *S* Fed solid diet. *P* Fed powder diet

Fourty-eight male C57 BL/6J mice were used in this study. The mice were kept under a 12 h light/dark cycle, with lights off from 20:00 to 8:00. They were randomly divided into the I/S group (Intact upper molar teeth/Solid diet feeding, n = 12), the E/S group (Extracted upper molar teeth/Solid diet feeding, n = 12), the I/P group (Intact upper molar teeth/Powder diet feeding, n = 12), and the E/P group (Extracted upper molar teeth/Powder diet feeding, n = 12) (Fig. 1). In the E/S and E/P group mice, the maxillary molar teeth on both sides were extracted at the age of 28 weeks, under general anesthesia with pentobarbital sodium (35 mg/kg, i.p. Somnopentyl, Kyoritsu Seiyaku Corporation, Tokyo, Japan). In contrast, the I/S and I/P group mice were anesthetized with pentobarbital sodium at the age of 28 weeks, but molar extraction was not performed. During 4-week (short term) or 16-week (long term) experimental periods, the I/S and E/S groups were fed chow pellets, whereas the I/P and E/P groups were fed a powder diet containing same ingredients as solid diet. The body weight of mice were measured once per week during the experimental period.

### Evaluation of memory ability

At 32 or 44 weeks of age (4 and 16 weeks after the baseline = 28 weeks), a step-through passive avoidance test was used to evaluate memory abilities. The apparatus consisted of a light compartment (90 mm × 115 mm × 150 mm) and a dark compartment with a steel rod grid floor connected to a shock generator (140 mm × 175 mm × 150 mm). The compartments were divided by a wall with a guillotine door. This behavioral test consisted of an acquisition trial and a retention trial. In the acquisition trial, each mouse was put into the light compartment and allowed to explore freely. After 15 s, the guillotine door was opened, and the mouse was allowed to move to the dark compartment. The latency time until the mouse completely entered the dark compartment was measured. Then, the mice received a 3 mA foot shock for 3 s through the steel rod grid floor. After the acquisition trial, mice were returned to the cage. One day later, for the retention trial, mice were again put into the light room, and the latency time to enter the dark room was measured without any foot shock. The maximum cut-off latency time was set to 300 s.

### Real-time polymerase chain reaction (RT-PCR)

BDNF and TrkB mRNA expression in the hippocampus and hypothalamic areas were analyzed by RT-PCR. The hippocampus and hypothalamus were removed and quickly frozen in the liquid nitrogen to be stored at −80 °C. Total RNA was isolated from tissue with RNeasy Mini Kit (QIAGEN, Hilden, Germany) according manufacturer’s instructions. The total RNA concentration was corrected to 100 ng/μL with a Q5000 spectrophotometer (Tomy, Tokyo, Japan). First strand cDNA synthesis was carried out using SuperScriptIII Reverse Transcriptase (Invitrogen, Carlsbad, CA). PCR amplification was performed using a Stratagene Mx3000P QPCR system (Agilent Technologies, La Jolla, CA). The sequences of the primers used in this analysis were as follows: forward, 5′-CGACGACATCACTGGCTGACA-3′ and reverse, 5′-CCAAAGGCACTTGACTGCTGAG-3′ for BDNF; forward, 5′-CAAGAACGAGTATGGGAAGGATGAG-3′ and reverse, 5′-TTGGCGTGGTCCAGTCTTCATA-3′ for TrkB; and forward, 5′-GTAGACAAAATGGTGAAGGTCGGT-3′ and reverse, 5′-ACAATCTCCACTTTGCCACTGC-3′ for glyceraldehyde-3-phosphate dehydrogenase (GAPDH), as the internal control. The polymerase activation step was at 40 cycles of 95 °C for 10 s and 60 °C for 30 s, followed by 95 °C for 10 min. Results were analyzed following the 2^−ΔΔCt^ method using GAPDH.

### Quantification of hippocampal pyramidal cells

Sections were Nissl stained to identify pyramidal cells in the hippocampus and hypothalamus. The number of pyramidal cells with clear nuclei, cell bodies, and cell boundaries was counted in a blinded fashion by light microscopy in the CA1 (100 μm × 300 μm) and the CA3 (200 μm × 200 μm) regions, according to previously reported methods [9, 21].

### Statistical analysis

All values are presented as mean values ± standard errors of mean (SEM). In the passive avoidance test, Mann–Whitney’s *U* test was used. One-way and two-way ANOVAs were used for evaluation of molar loss and powder diet. When significant effects were detected, subsequent post hoc analyses were performed with Tukey’s post hoc test. P values of <0.05 were considered significant.

## Results

**Fig. 2:**
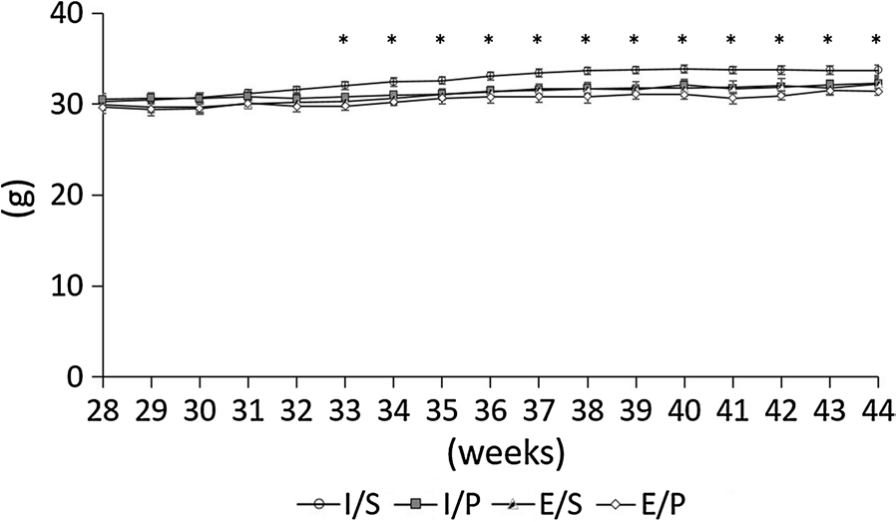
Body weight. During 28–31 week period, body weight were not significantly different among all group (n = 12). After 32 weeks, the weight of the E/P group was significantly lower than that of the I/S group. *P < 0.05, E/P vs I/S

Body weights were not significantly different during the 28- to 31-week period among all groups (Fig. 2). However, after 32 weeks of age, the weight of the E/P group was significantly lower than that of the I/S group.

### Passive avoidance test

#### Four-week period

**Fig. 3:**
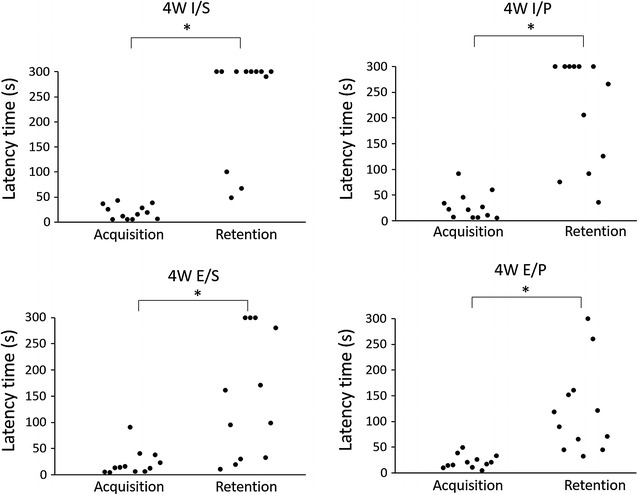
Latency time after 4-week period. The latency time of the retention trial was significantly longer than that of the acquisition trial in all groups (n = 12)

We performed a passive avoidance test at 32 weeks of age to analyze the memory ability. As shown in Fig. 3, the latency time of the retention trial was significantly longer than that of the acquisition trial in all groups. These findings indicate that the no group of mice showed impaired memory ability. Additionally, the latency times were no statistically different between the groups in acquisition trial. In retention trial, however, the latency times of molar extracted group were shorter than molar intact group.

#### Sixteen-week period

**Fig. 4:**
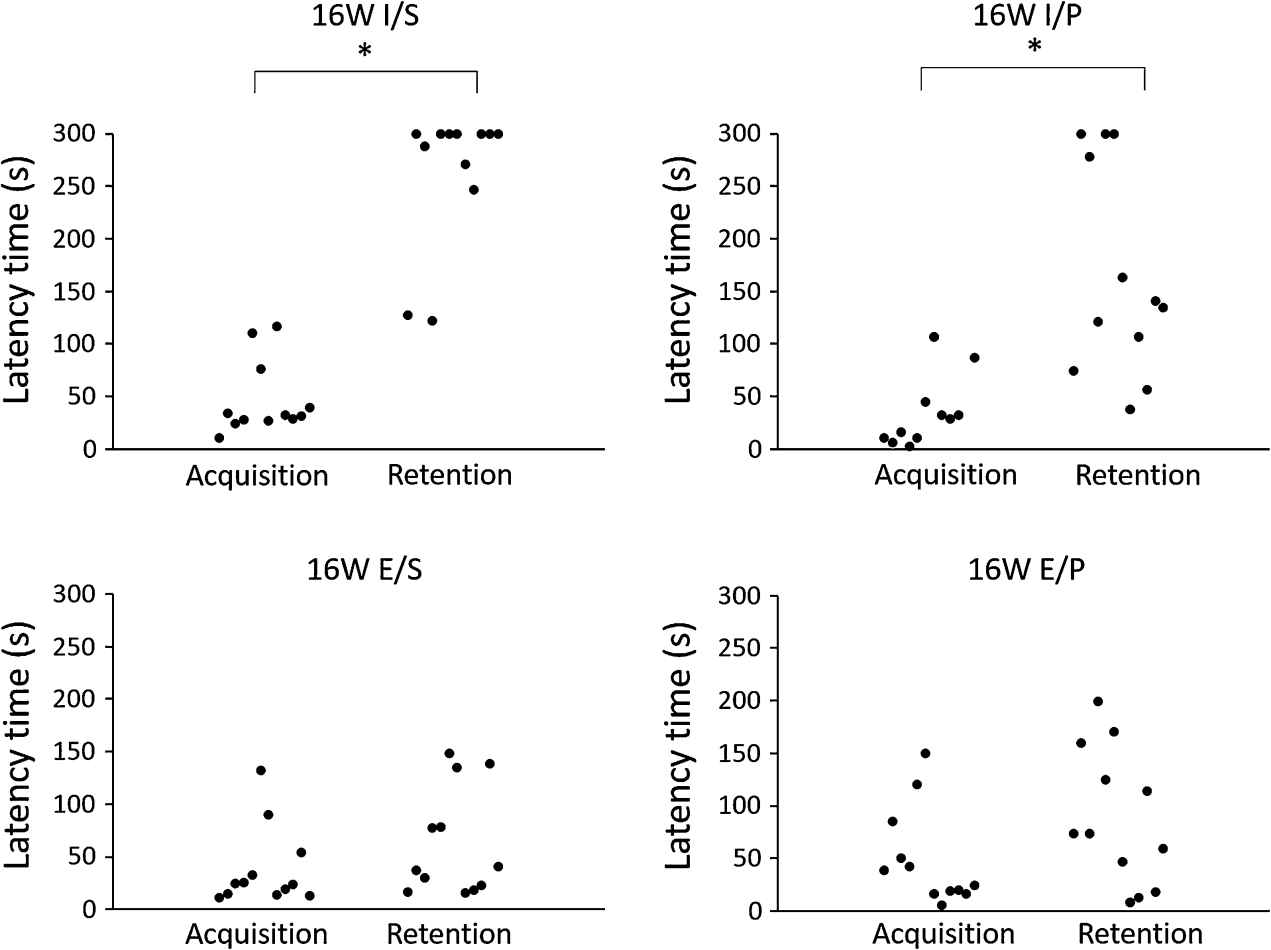
Latency time after 16-week period. The latency time of the retention trial was significantly longer than that of the acquisition trial in the I/S and I/P groups (n = 12). Meanwhile, the latency time was not significantly different between the acquisition and retention trials in the E/S and E/P groups (n = 12)

The results of the passive avoidance test at 44 weeks of age are shown in Fig. 4. The latency time of the retention trial was significantly longer than that of the acquisition trial in the I/S and I/P groups. Meanwhile, the latency time was not significantly different between the acquisition and retention trials in the E/S and E/P groups. These results indicate that the memory ability was impaired in the E/S and E/P groups. As with four-week period, the latency times of acquisition trial were no significantly different between the groups. However, the latency time of molar extracted group and powder diet group were shorter than molar intact group and solid diet group.

### Pyramidal cells

#### Four-week period

**Fig. 5:**
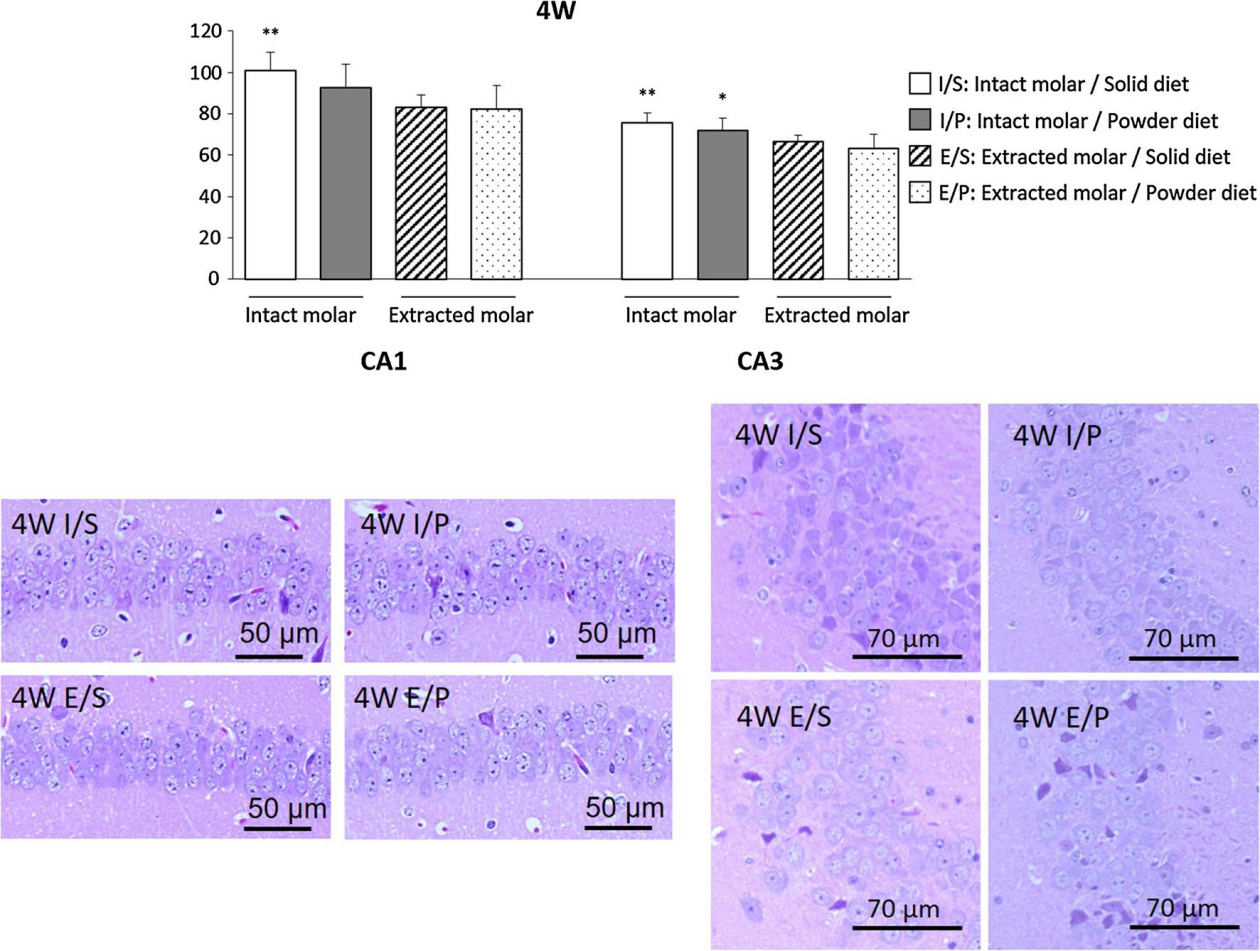
Pyramidal cell in 4-week period. In CA1 and CA3 region, extracted molar mice had fewer pyramidal cells than intact molar mice. The number of pyramidal cells were similar between solid and powder diet mice. Two-way ANOVA were used to compare the groups. *F*(1, 20) = 12.52, P < 0.01 for molar loss; *F*(1, 20) = 1.36, P = 0.26 for powder diet; *F*(1, 20) = 0.91, P = 0.35 for molar loss × powder diet in CA1. *F*(1, 20) = 15.79, P < 0.01 for molar loss; *F*(1, 20) = 2.57, P = 0.12 for powder diet; *F*(1, 20) = 0.01, P = 0.94 for molar loss × powder diet in CA3. **, *P < 0.01, 0.05 vs respective extracted molar mice at same diet. Data represent mean ± SEM

Figure 5 shows representative images of CA1 and CA3 region. Histological analysis showed that molarless mice had fewer pyramidal cells than intact molar mice both CA1 and CA3 region. The number of pyramidal cell in CA1 and CA3 were similar between solid and powder diet mice. Additive effect of powder diet on molar loss did not reach a significant difference.

#### Sixteen-week period

**Fig. 6:**
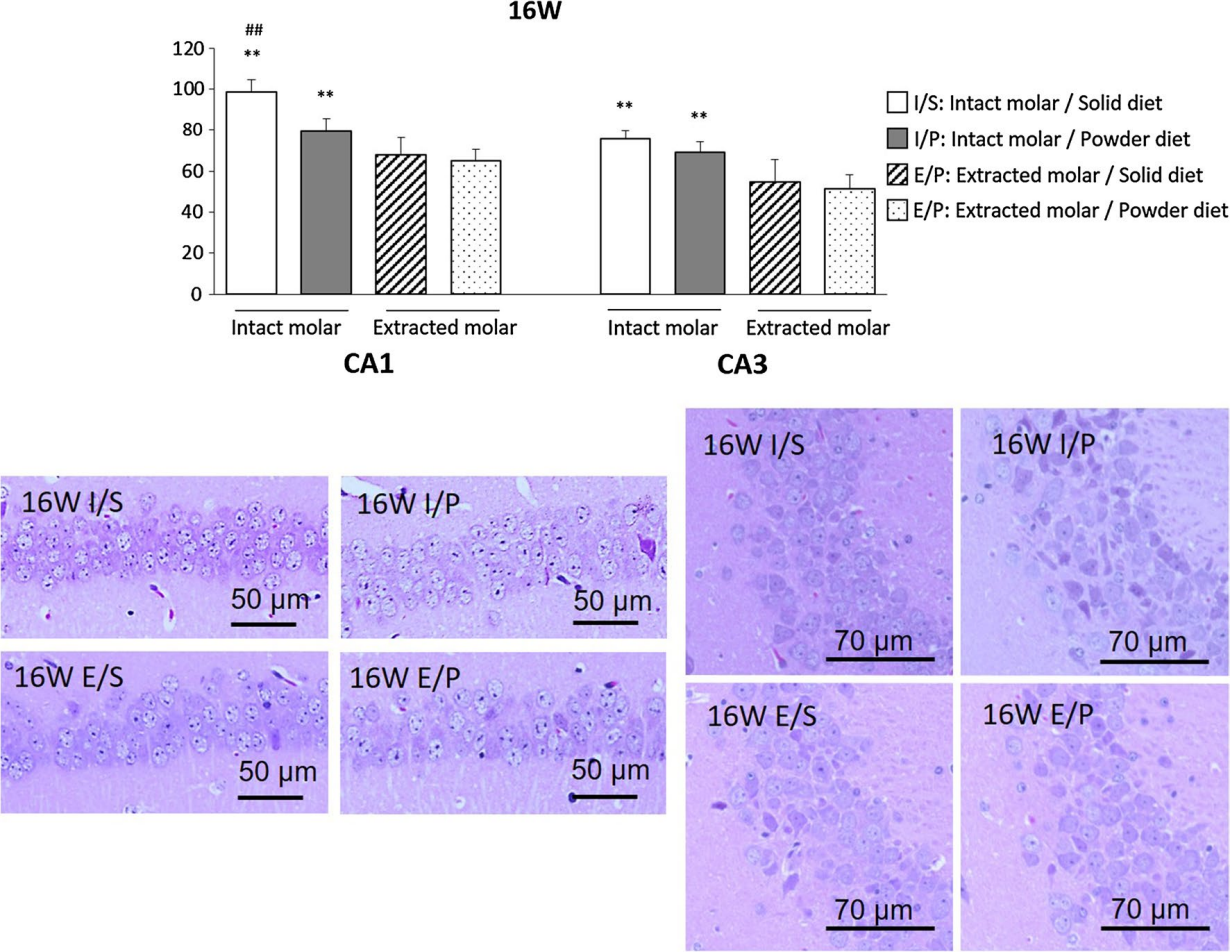
Pyramidal cell in 16-week period. In CA1 and CA3 region, extracted molar mice had fewer pyramidal cells than intact molar mice. In CA1 region, powder diet mice had fewer than solid diet mice. Two-way ANOVA were used to compare the groups. *F*(1, 20) = 70.93, P < 0.01 for molar loss; *F*(1, 20) = 42.75, P < 0.01 for powder diet; *F*(1, 20) = 8.72, P < 0.01 for molar loss × powder diet in CA1. *F*(1, 20) = 16.57, P < 0.01 for molar loss; *F*(1, 20) = 3.13, P = 0.09 for powder diet; *F*(1, 20) = 0.35, P = 0.56 for molar loss × powder diet in CA3. **P < 0.01 vs respective extracted molar mice at same diet, ##P < 0.01 vs powder diet at intact molar mice. Data represent mean ± SEM

Figure 6 shows the representative image of CA1 and CA3 region. The molarless mice had fewer pyramidal cells compared to molar-intact mice in CA1 and CA3 regions. Although the number of pyramidal cells in CA3 were similar between solid and powder diet mice, powder diet mice had fewer pyramidal cells than solid diet mice in CA1. Additive effect of molar loss and powder diet reached a significant difference in CA1, but not CA3.

### mRNA levels

**Fig. 7:**
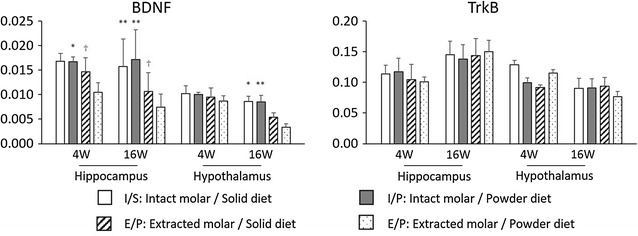
Influence of molar loss and powder diet on BDNF and TrkB mRNA levels in the hippocampus and hypothalamus of 4 and 16 week periods. mRNA levels were determined by RT-PCR and normalized relative to the level of GAPDH mRNA detected in each sample. Two-way ANOVA were used to compare the groups. 4 W Hippocampus BDNF; *F*(1, 20) = 4.36, P = 0.05 for molar loss, *F*(1, 20) = 1.19, P = 0.29 for powder diet, *F*(1, 20) = 1.08, P = 0.31 for molar loss × powder diet. 4 W Hippocampus TrkB; *F*(1, 20) = 0.48, P = 0.50 for molar loss, *F*(1, 20) = 0.00, P = 0.99 for powder diet, *F*(1, 20) = 0.04, P = 0.85 for molar loss × powder diet. 16 W Hippocampus BDNF, *F*(1, 20) = 46.33, P < 0.01 for molar loss, *F*(1, 20) = 0.71, P = 0.41 for powder diet, *F*(1, 20) = 4.52, P < 0.05 for molar loss × powder diet. 16 W Hippocampus Trk B; *F*(1, 20) = 2.82, P = 0.11 for molar loss, *F*(1, 20) = 0.18, P = 0.67 for powder diet, *F*(1, 20) = 17.86, P < 0.01 for molar loss × powder diet. 4 W Hypothalamus BDNF; *F*(1, 20) = 0.53, P = 0.48 for molar loss, *F*(1, 20) = 0.14, P = 0.71 for powder diet, *F*(1, 20) = 0.05, P = 0.82 for molar loss × powder diet. 4 W Hypothalamus TrkB; *F*(1, 20) = 0.05, P = 0.82 for molar loss, *F*(1, 20) = 0.00, P = 0.98 for powder diet, *F*(1, 20) = 0.08, P = 0.78 for molar loss × powder diet. 16 W Hypothalamus BDNF; *F*(1, 20) = 16.71, P < 0.01 for molar loss, *F*(1, 20) = 1.13, P = 0.30 for powder diet, *F*(1, 20) = 0.86, P = 0.36 for molar loss × powder diet. 16 W Hypothalamus TrkB; *F*(1, 20) = 0.14, P = 0.71 for molar loss, *F*(1, 20) = 0.33, P = 0.57 for powder diet, *F*(1, 20) = 0.38, P = 0.54 for molar loss × powder diet. **, *P < 0.01, 0.05 vs respective extracted molar mice at same diet, ^†^P < 0.05 vs powder diet at extracted molar mice

BDNF and TrkB mRNA levels in hippocampus and hypothalamus are shown in Fig. 7.

#### Four-week period: hippocampus

BDNF mRNA levels of extracted molar mice were significantly reduced than intact molar mice in powder diet. Also, BDNF levels of powder diet mice were significantly reduced than solid diet in extracted mice. Two-way ANOVA analysis revealed no significant main effect of molar loss, diet, or interaction between molar loss and powder diet on BDNF level. Regarding TrkB expression, analysis revealed that there was no significant difference between the effect of molar loss, powder diet, or interaction between molar loss and diet on TrkB mRNA level.

#### Four-week period: hypothalamus

There were no significant main effect of molar loss, power diet, or interaction between molar loss and powder diet.

#### Sixteen-week period: hippocampus

BDNF mRNA levels of extracted molar mice were significantly reduced than intact molar mice in respective diet. Also, BDNF mRNA levels of powder diet mice were reduced than solid diet mice in extracted mice. There was a significant main effect of molar loss, but there were no significant main effect of powder diet on BDNF and TrkB. Additionally, BDNF showed significant interaction between molar loss and powder diet.

#### Sixteen-week period: hypothalamus

There was a significant main effect of molar loss only BDNF, but no significant main effects of diet and no significant interaction.

## Discussion

Human studies have reported that masticatory imbalances could impair memory ability or reduce the number of brain neurons [4, 22]. Animal studies suggested that molar loss or soft diet in younger periods had a harmful effect on normal growth [8, 17]. However, few studies observed the effect of a combination of molar loss and powder diet in adult mice. In clinical settings, when patients have lost teeth due to caries or periodontitis, they could not masticate effectively. As a result, molar loss may lead to intake of a soft diet instead of a hard diet. Namely, most patients without healthy dentition eat soft diets. However, in previous reports, mainly younger or transgenic mice were used to analyze the effects of molar loss on brain neurons or memory ability [23, 24]. Accordingly, to observe each effect, tooth loss and powder diet, we chose normal mice, not transgenic mice. Additionally, we want to observe the effect of molar loss and soft diet in adulthood. It is well known that body weight of C57BL/6J mice is increased until around 20 weeks old. After 20–28 weeks, increase of body weight is stable. Therefore, we have considered that molar removal at 28 weeks old was proper to eliminate the effect of body changing. Thus, our experimental design, using an adult molar loss model mouse strain in conjunction with powder diet feeding, was considered adequate for simulation of an actual clinical setting. We also hypothesized that the conjunction of molar loss and powder diet might enhance the effect of each factor.

In this study, the body weight between the groups did not significantly differ during the first 4 weeks. However, body weight was significantly different between the I/S and E/P groups after 32 weeks of age. This finding is inconsistent with some reports that showed different results. One study showed that the body weight of the molar loss or soft diet group was significantly lower than that of the control group from baseline [10, 25]. Another study reported that the body weight of solid diet and liquid diet mice was not significantly different [17]. Because the mice in our study were older than those in other studies, the effect of molar loss or powder diet on mouse body weight might not be significant in the first 4-week period. However, continuous loss of teeth with a powder diet might affect body change. BDNF and its receptor, TrkB, are expressed in hypothalamus associated with satiety and locomotor activity [26]. BDNF has important role of weight-control or eating behavior. Our results suggested that the combination of molar loss and powder diet could impair weight control or energy homeostasis.

The hippocampus has a crucial role in memory ability. Decrease and atrophy of pyramidal cells in the CA1 and CA3 regions have been associated with the deterioration of memory ability [27]. Therefore, we assessed the number of pyramidal cells in the CA1 and CA3 regions of the hippocampus. In the hypothalamus, which is known to be involved in feeding-related behavior or weight control, neurogenesis has been shown to occur constitutively [26, 28], and Patten et al. [29] observed a significant reduction in cellular proliferation in the hypothalamus of soft diet-fed animals. We also observed that molar loss caused a decrease in BDNF mRNA expression. Molar loss may therefore lead to breakdown of BDNF homeostasis, possibly by inhibition of feeding-related behavior. Some studies reported that stress involving molar loss or occlusal disharmony may impair learning and memory ability [10, 30]. However, the persistence of the effects of stress over long periods is unknown. Further study is needed to fully determine the mechanism of these effects.

Many behavioral tests such as eight arms radial maze or water maze task have been used to assess the memory ability [41, 42]. These tests need locomotor activity which affects BDNF expression [43]. To remove the effect of locomotor activity, we performed step-through passive avoidance test which is regarded as simpler task than other behavioral test. At the 4-week period, none of the groups demonstrated memory impairment. However, both the molar loss groups, regardless of diet texture demonstrated memory impairment at 16-week period. These results suggest that short periods of molar loss might not cause memory impairment in adult mice, but in long periods it might cause cognitive impairment. We also analyzed the data in not only each group but also between the group. The differences of latency time were found in retention trial, not in acquisition trial. This result indicates that molar extracted and powder affect the latency time in retention trial.

These observations also suggest that molar loss has a greater effect on memory impairment than powder diet feeding. The interactions or connections between brain synapses might be immature at a young age, therefore the decreased oral sensory stimuli, resulting from the extraction or reduction of molar teeth, could influence memory ability. In aged mice, however, because the interactions between brain synapses were already mature, a short-term decrease in sensory stimuli might not impair memory ability. Some studies reported that either extraction of molar teeth impaired learning ability in aged mice at approximately the 4-week period [10, 31]. This was presumably due to the use of a transgenic mouse model in these studies. The maturation course of senescence-accelerated or other transgenic mice may be different from normal mice.

Meanwhile, the molar loss groups showed decreased number of pyramidal cells in the hippocampal CA1 and CA3 regions at the 4- and 16-week time points. There were also fewer pyramidal cells in the powder diet group than in the hard diet group. Moreover, the mRNA levels of BDNF and TrkB in the molar loss groups tended to decrease compared with the intact groups. A significant main effect of molar loss and no significant main effect of powder diet were found, but a significant interaction between molar loss and powder diet was observed at 16-week period. The effect of powder diet was less than molar loss, but long-term conditions of molar loss and powder diet might lead to decreased BDNF and TrkB mRNA levels and pyramidal cells, resulting in cognitive impairment. At 4 weeks post-molar removal, molar removal was associated with a decrease in pyramidal cells and BDNF in the hippocampus, but no corresponding changes in memory. There may be a time lag between exhibiting memory impairment and structural brain changes.

The limitation of our study should be considered. Some studies reported the correlation between stress and cognitive impairment or BDNF [32, 33]. The molarless condition increases blood corticosterone levels and impairs spatial memory ability [30]. Since, stress and an increase in corticocteron levels are known to inhibit cell proliferation and overall neurogenesis in the hippocampus [34–36], it has been suggested that the reduction of cell proliferation observed in animal fed a soft diet might also involve an increase in plasma level of corticosterone [37–39]. Moreover, soft-diet feeding enhances oxidative stress, which leads to oxidation and a decrease in the release of dopamine in the hippocampus of rats [40]. However, inconsistent results that there were no significant differences between rats on the liquid or soft diet have been observed [29]. Our study did not perform the confirmation of stress. Thereby, we could not show how the stress caused by molar loss and powder diet lead to the change of BDNF and TrkB mRNA levels. Further studies are required to clarify these hypotheses.

## Conclusion

The results of this study suggest that, in adult mice, the effects of molar loss and powder diet are different, and molar loss may have a greater long-term effect on memory ability than powder diet does. Thus, we could suggest that the maintenance of teeth and mastication might be important for prevention of cognitive impairment.

## Abbreviations

BDNF: brain-derived neurotrophic factor
IRS-1/2: insulin receptor substrate-1
I/S: intact upper molar teeth/Solid diet feeding
E/S: extracted upper molar teeth/Solid diet feeding
I/P: intact upper molar teeth/Powder diet feeding
E/P: extracted upper molar teeth/Powder diet feeding
Akt: protein kinase B
TrkB: tyrosine kinase B
RT-PCR: real-time polymerase chain reaction
GAPDH: glyceraldehyde-3-phosphate dehydrogenase
SEM: standard errors of mean
